# Transcriptional profiling reveals *H.pylori*-associated genes induced inflammatory cell infiltration and chemoresistance in gastric cancer

**DOI:** 10.3389/fimmu.2025.1592558

**Published:** 2025-05-30

**Authors:** Jinshui Tan, Zhengxin Wu, Yuankun Liu, Wei Wang, Wenjuan Qin, Guangchao Pan, Yubo Xiong, Jingsong Ma, Jiabao Zhao, Huiwen Zhou, ZhengJin Liu, Haijie Lu, Huiqin Zhuo, Xuehui Hong

**Affiliations:** ^1^ Department of Gastrointestinal Surgery, Zhongshan Hospital of Xiamen University, School of Medicine, Xiamen University, Xiamen, China; ^2^ Department of Gastrointestinal Surgery, Xiamen Municipal Key Laboratory of Gastrointestinal Oncology, Xiamen, China; ^3^ Department of Radiology, The First Affiliated Hospital of Zhejiang Chinese Medical University, Hangzhou, China; ^4^ Department of Gastroenterology, The National Key Clinical Specialty, Clinical Research Center for Gut Microbiota and Digestive Diseases of Fujian Province, Key Laboratory for Intestinal Microbiome and Human Health of Xiamen, Zhongshan Hospital of Xiamen University, School of Medicine, Xiamen University, Xiamen, China; ^5^ Department of Radiation Oncology, Zhongshan Hospital of Xiamen University, School of Medicine, Xiamen University, Xiamen, China; ^6^ Department of Pathology, Zhongshan Hospital of Xiamen University, School of Medicine, Xiamen University, Xiamen, China

**Keywords:** gastric cancer, *H. pylori*, tumor microenvironment, HspB2, ACSM5, eosinophils

## Abstract

**Background:**

*H. pylori* infection is closely associated with the tumor microenvironment (TME) in gastric cancer (GC), yet its underlying mechanism is elusive. Hence, it is imperative to explore the microenvironment and drug resistance arising from *H. pylori* to enhance therapeutic strategies for GC.

**Methods:**

Employing transcriptional bioinformatics, we computed a *H. pylori*-associated prognostic index (HPI) using datasets from TCGA and GSE62254 containing ACSM5 and HSPB2 gene expression. We assessed IC50 values for anticancer drugs and immune cell infiltration to evaluate the therapeutics and TME based on the HPI. Further, we validated the transcriptional profiling findings by examining drug sensitivity transfected with siACSM5 and siHSPB2 and analyzing scRNA-seq data and clinical patient samples.

**Results:**

ACSM5 and HSPB2 were identified as correlates of *H. pylori* infection in GC. Significantly, we established the *H. pylori*-associated prognostic index (HPI) and found that a high HPI was linked with a worse prognosis. Classification based on the HPI indicated an enhanced infiltration of tumor microenvironment cells and resistance to anti-tumor drugs.

**Conclusion:**

The HPI, reflecting newly identified and complementary biomarkers, correlated with the TME and could accurately project chemoresistance and an altered immune cell distribution in GC patients, thus providing clinical guidance on therapeutic interventions.

## Introduction

Gastric cancer (GC) ranks among the most prevalent cancers and is a leading cause of cancer-related mortality globally (1). Most patients are already in the advanced stage of GC when they are first diagnosed, which leads to a poor prognosis (2). *Helicobacter pylori* (*H. pylori*), the main environmental contributor to GC, is implicated in nearly 90% of its newly diagnosed cases (2, 3). *H. pylori* invades the host gastric mucosa, causing epithelial damage and heightening GC risk through mechanisms involving flagella, adhesin, and cytotoxins (VacA and CagA) (4). During GC progression post-*H. pylori* infection, tumor cells interplay with a complex and dynamic tumor microenvironment (TME) (5), mainly including tumor cells, immune and stromal cells, tumor vasculature and metabolic byproducts (6). The TME implicated cancer metabolism, angiogenesis, metastasis and chemoresistance, and notably influences immunomodulatory interactions (7).

Recent focus on *H. pylori*’s impact in the TME has spanned DNA damage, oncogenic signaling, and immune regulation. Studies have unearthed that *H. pylori* infection triggers significant inflammation, leading to cellular hypoxia and metabolic disruption associated with the TME (8). Inflammatory cells in the gastric mucosa post-infection, such as macrophages and neutrophils, producing an excess of reactive oxygen species and DNA damage (9). P53 mutations, a pivotal TME regulator that promotes angiogenesis via fibroblast activation and VEGF secretion (10, 11). Furthermore, *H. pylori* activates HIF-1α through the PI3K/AKT/mTOR pathway to foster inflammatory factor production, cancer cell invasion, and alter traditional radiotherapy and chemotherapy responses (12).

Despite insights into *H. pylori*’s influence on the TME, the correlation between *H. pylori* infection status and GC patient prognosis remains unclear. Therefore, to reveal the role of *H. pylori* infection in the progression and outcome of GC patients, our study utilized bioinformatics to flag potential TME-related differentially expressed genes (DEGs) in *H. pylori*-positive GC and compute the *H. pylori*-associated prognostic index (HPI). The HPI-based assessment revealed potential contributions of drug resistance and immune infiltration in *H. pylori*-infected GC to adverse prognoses.

## Materials and methods

### Data collection

We sourced raw RNA-seq data (FPKM normalized) and patient follow-up information from stomach adenocarcinoma (STAD) dataset in The Cancer Genome Atlas (TCGA) (n=348) (https://www.cancer.gov/) and RMA-normalized microarray gene expression data of GSE62254 (n=300) from GEO (https://www.ncbi.nlm.nih.gov/geo/).

The processed expression matrices of scRNAseq were download from OMIX001073 (13) (https://ngdc.cncb.ac.cn/omix/release/OMIX001073). Three *H. pylori* negative cases and three positive cases were included in this study. Seurat package (version 4.0) was used for cell normalization and regression to obtain the scaled data. Harmony is used to integrate data. PCA was constructed to identify high variable genes based on the scaled data and top 15 principals were used for tSNE construction. we calculated the cluster marker genes by FindAllMarkers function with wilcox rank. And the clusters of same cell type were selected for re-tSNE analysis and annotation.

### Analysis of TME scores

We employed the “estimate” R package (14) for immune/stromal/estimate scoring. The optimal cutoff point was calculated and the Kaplan–Meier survival curves were plotted via the “survminer” package. Pearson method was used to assess correlations between *H. pylori* infection status and TME scores.

### Weighted gene co-expression network analysis and differentially expressed gene analysis

WGCNA (15) helped identify co-expressed gene modules related to *H. pylori* infection and immune/stromal/estimate scores. The analysis, including functions for network construction, module detection, gene selection, calculations of topological properties, data simulation and visualization was based on the “WCGNA” R package. DEG between groups were identified through Wilcoxon test. Results from DEG analysis and WGCNA formed the input for LASSO-penalized Cox regression analysis.

### LASSO-penalized Cox regression analysis and development *H. pylori*-associated prognostic index development

LASSO-penalized Cox regression analysis is based on the penalty method for variable selection of sample data. By compressing the original coefficients, the small coefficients are compressed to 0, thus directly discarding unimportant variables (16). Analysis facilitated the elimination of lesser impactful genes, allowing us to compute a prognostic risk value based on the remaining DEGs and construct the *H. pylori*-associated prognostic index (HPI) as follows: HPI =∑β_i_*Exp_i_, with β_i_ being each gene’s coefficient, and Exp_i_ is gene expression value. The function and performance of the model, including the determination of the best cutoff value and Kaplan–Meier survival analysis, was utilized by the “survminer” package.

### Prediction of TME signatures and therapeutic sensitivity

The immune cell and stromal cell infiltrating levels were assessed using the “CIBERSORT” (17), “EPIC”, “TIMER” (18) and “XCELL” (19) algorithms in R. Additionally, HPI’s predictive capability for chemotherapy/targeted therapy response. was examined by determining the 50% inhibitory concentration (IC50) value with the “pRRophetic” algorithm (20).

### Sample collection

Gastric tumor tissues with or without *H.pylori* infection(n = 10, respectively) were collected during surgical resection at the Affiliated Zhongshan Hospital of Xiamen University (Fujian Province, China) between 2022 and 2023. This study was approved by the Ethics Review Committee of the Affiliated Zhongshan Hospital of Xiamen University(xmzsyyky-2022-160).

### Cell lines and culture conditions

In this study, the GC cell lines HGC-27 and MKN45 were purchased from Cobioer Biotechnology Company (Jiangsu, China). They were both maintained in RPMI 1640 (Procell, China) supplemented with 10% fetal bovine serum (Gibco, USA) at 37°C in a humid environment containing 5% CO2 and 95% air.

### Cell viability assessment and drug sensitivity assessment

To assess the cytotoxic effects of 5-fluorouracil and paclitaxel, vector-, siHSPB2- and siACSM5-transfected HGC-27 and MKN45 cells were exposed to 5-Fu (50 μM) and PTX (2.5 nM) for 24 h. Next, CCK-8 reagent (10 μl/well) (Topscience, China) was added and incubated for an additional 2 h at 37°C. The absorbance was detected at 450 nm in a Bio-Rad microplate reader (Bio-Rad, CA, USA).

### Immunohistochemistry

Tissue microarrays were obtained from Shanghai OUTDO Biotechnology Co., Ltd. (HStmA180Su30, Shanghai, China). The studies were conducted in accordance with the International Ethical Guidelines for Biomedical Research Involving Human Subjects (CIOMS), and the research protocols were approved by the Ethics Review Committee of the Affiliated Zhongshan Hospital of Xiamen University (xmzsyyky-2022-160). After deparaffinization and rehydration, gastric enzyme (Maxim, DIG-3009, Fujian, China) was used to repair antigens. Then, the Ultrasensitive SP kit (KIT-9730, Maxim, Fujian, China) was utilized to block endogenous peroxidase activity in the tissue microarray for subsequent immunohistochemistry analysis. The following antibodies were applied at 4°C overnight: anti-ACSM5 (1:4000, Proteintech, 67334–1-lg, Hubei, China) and anti-HSPB2 (1:500, Proteintech, 21755–1-AP, Hubei, China). Later, the secondary antibody in the Ultrasensitive SP kit was utilized. An enhanced DAB chromogenic kit (Maxim, DAB-2032, Fujian, China) was employed to complete the detection. Finally, hematoxylin and eosin staining were performed on the tissue microarray, and the samples were mounted and observed under a microscope.

### Immunofluorescence

According to the manufacturer’s instructions, gastric enzyme (Maxim, DIG-3009, Fuzhou, China) was used for antigen repair, similar to the protocol for IHC. The Ultrasensitive SP kit (Maxim, KIT-9730, Fujian, China) was employed to restore endogenous peroxidase activity for immunofluorescence experiments. The following antibodies were applied at 4°C overnight: CD80 (1:250, ab270113, Abcam, Cambridge, USA) and iNOS (1:200, ab3523, Abcam, Cambridge, USA). The corresponding secondary antibodies with fluorescence were applied next: goat anti-rabbit IgG (1:1000, ab150077, Abcam, Cambridge, USA). After three washes with PBS, the slices were stained with DAPI to detect nuclei (1:1000, F6057, Sigma–Aldrich) and visualized with confocal Microscope.

### siRNA and transfection

The Specific siRNAs against human HSPB2 and ACSM5 were purchased from the Public Protein/Plasmid Library (Nanjing, China). They were transfected into cells by utilizing Lipofectamine 3000 Reagent (Invitrogen) according to the directions. The transfection efficiency was examined by western blotting after 48 h of transfection.

### Western blotting

The HGC-27 and MKN45 GC cell lines were harvested and lysed at 4°C. Quantitative analysis of protein was performed with the Pierce BCA Protein Assay kit (23227, Thermo Scientific, Shanghai China). Proteins were separated by SDS–PAGE, and the following primary antibodies and secondary antibodies were applied based on the instructions: ACSM5 (1:500, 67334–1-lg, Proteintech, Hubei, China), HSPB2 (1:500, 21755–1-AP, Proteintech, Hubei, China), β-actin (1:1000, Cat#3700, CST, USA), anti-mouse secondary antibodies (1:1000, 1706516, Bio-Rad, Hercules, CA) and anti-rabbit secondary antibody (1:1000, ab150077, Abcam, Cambridge, USA). Enhanced chemiluminescence (Bio-Rad, USA) was utilized for detection.

### Flow cytometry

The tumor tissues and adjacent normal tissues were cut into small pieces of approximately 1 mm^3^ and digested with trypsin-EDTA solution (2122153, BI, Israel) for 10 mins at 37°C. The digestion was subsequently terminated with Hank’s buffer (1803241, Procell, China). Then, the digested tissues were incubated for 1 h at 37°C in Hank’s buffer containing type IV collagenase (2357210, Gibco, USA), hyaluronidase (37326-33-3, Sigma-Aldrich, USA) and dispase (2309419, Gibco, USA). The dissociated cell suspensions were ground and filtered through an 80 μm cell filter (22131209, Biosharp, China), and red cells were lysed with ACK lysis buffer (R1010, Solarbio, China) to obtain a single cell suspension. The cells were stained with anti-human CD45 (1:200, 2317050, clone H130, Invitrogen, USA), anti-human CD11b (1:200, 2191966, clone ICRF44, Invitrogen, USA), anti-human CD15 (1:200, 301904, clone H198, Biolegend, USA), anti-human CD16 (1:200, 302012, clone 3G8, Biolegend, USA), anti-human CD273 (1:200, 316718, clone 5G8, Biolegend, USA), and anti-human siglec 8 (1:200, 347104, clone 7C9, Biolegend, USA) antibodies and fixable viability dye (1:200, 2365395, Invitrogen, USA) cocktails for 30 min. The stained cells were analyzed by the Fortessa-X20 (BD).

To evaluate drug sensitivity, the vector, siHSPB2 and siACSM5 cell lines of HGC-27 and MKN45 cells were exposed to 5-fluorouracil (50 μM) and Paclitaxel (2.5 nM) for 24 h. Next, the cells were digested with trypsin-EDTA solution for 2 min at 37°C. Harvested cells were stained with the Annexin V/PI apoptosis detection kit (BD Pharmingen, USA) for 10 min at room temperature in the dark according to the manufacturer’s protocols and then measured on the CytoFlex S (BD, USA). Three replicate experiments were performed to analyze apoptosis levels.

### Statistical analysis

For bioinformatic analysis, R software (version 4.0.4) was utilized. Correlation coefficients with absolute values greater than 0.2 and/or p< 0.05 were deemed to indicate statistical significance. For external experimental verifications, Wilcoxon test was utilized to assess the significance of the differences between different groups. GraphPad Prism 8.0.1 software was employed for statistical analysis.

## Results

### Comparison of TME scores between *H. pylori* positive and negative GC patients

*H. pylori* infection is a well-known driver of alterations in the tumor microenvironment, precipitating chronic inflammation in gastric cancer (21, 22). Microenvironment cell infiltration can be assessed according to the expression levels of relevant molecules in immune and stromal cells, and this approach is widely used to predict changes in the TME (23, 24). To assess whether TME composition differs in patients with *vs*. without *H. pylori* infection, we evaluated the TME scores, including stromal, immune, and estimate scores), using the “estimate” R package across 348 GC samples in the TCGA database. Tumor stromal scores ranged from -1856.53 to 2051.31, immune scores from -1049.88 to 3136.08, and ESTIMATE scores from -2460.62 to 4888.88. TME scores showed significantly distributional disparities between the *H. pylori^+^* and *H. pylori^-^* GC patients (**Supplementary Figure 1A**). Notably, *H. pylori^+^* patients exhibited higher TME scores than their *H. pylori* ^-^ counterparts, suggesting an intricate microenvironment in *H. pylori^+^* subgroup. Subsequent analysis revealed that patients with elevated stromal scores experienced poorer outcomes (*p*=0.009, **Supplementary Figure 1B**). Despite the lack of survival differences between the immune score and the estimate score groups (**Supplementary Figures 1C, D**), higher scores in these categories tend to correlate with poorer prognosis.

### Identification of DEGs related to *H. pylori* infection in the GC TME

**Figure 1:**
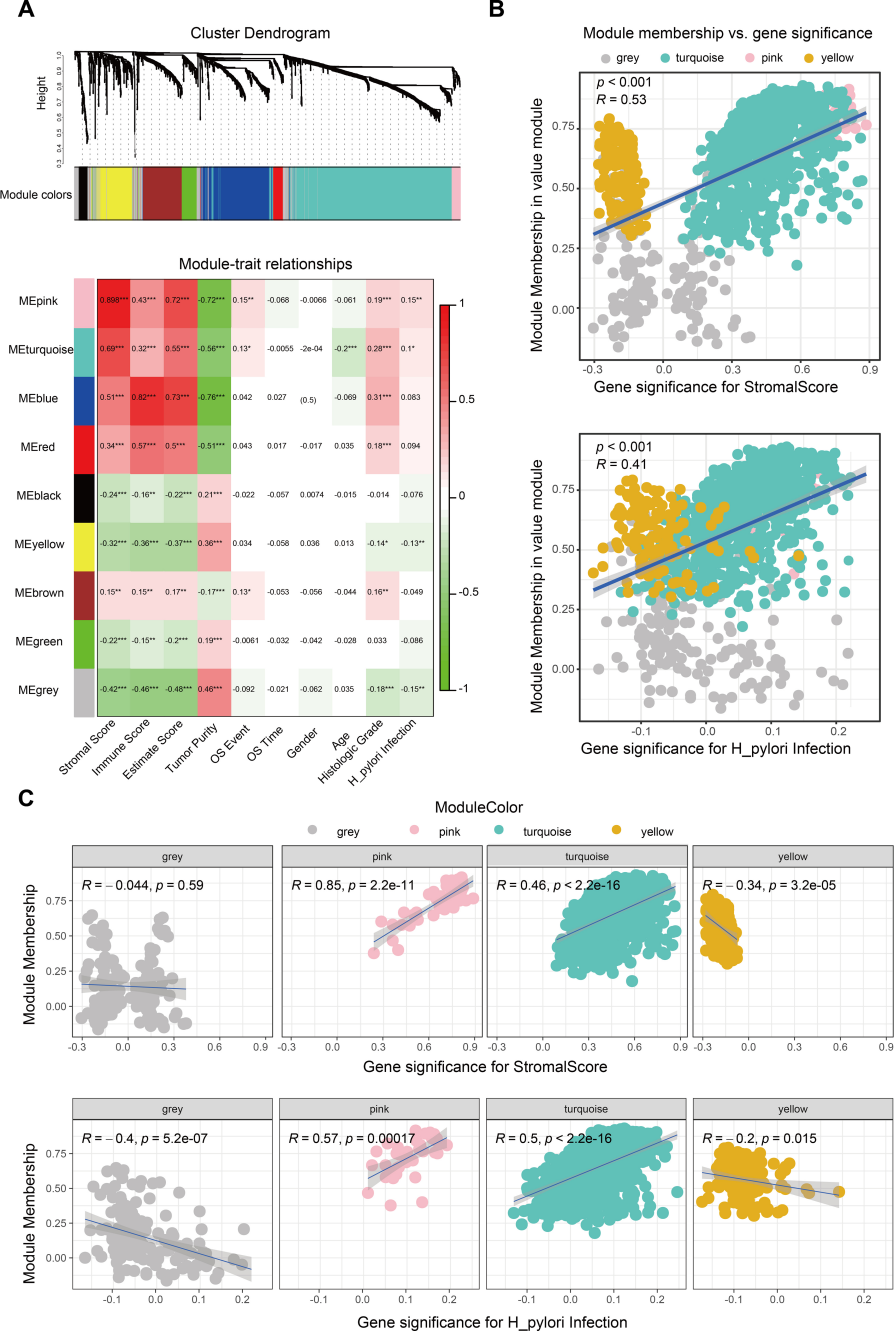
Identification of the *H*. *pylori*-associated TME module. **(A)** WGCNA to screen the significant gene modules associated with the TME and *H*. *pylori* infection. The gray, pink, turquoise and yellow modules were finally selected. **(B)** Correlation between significant module membership and stromal scores and *H. pylori* infection. **(C)** Correlation between the single significant module membership (gray, pink, turquoise and yellow module) and stromal scores and *H*. *pylori* infection.

**Figure 2:**
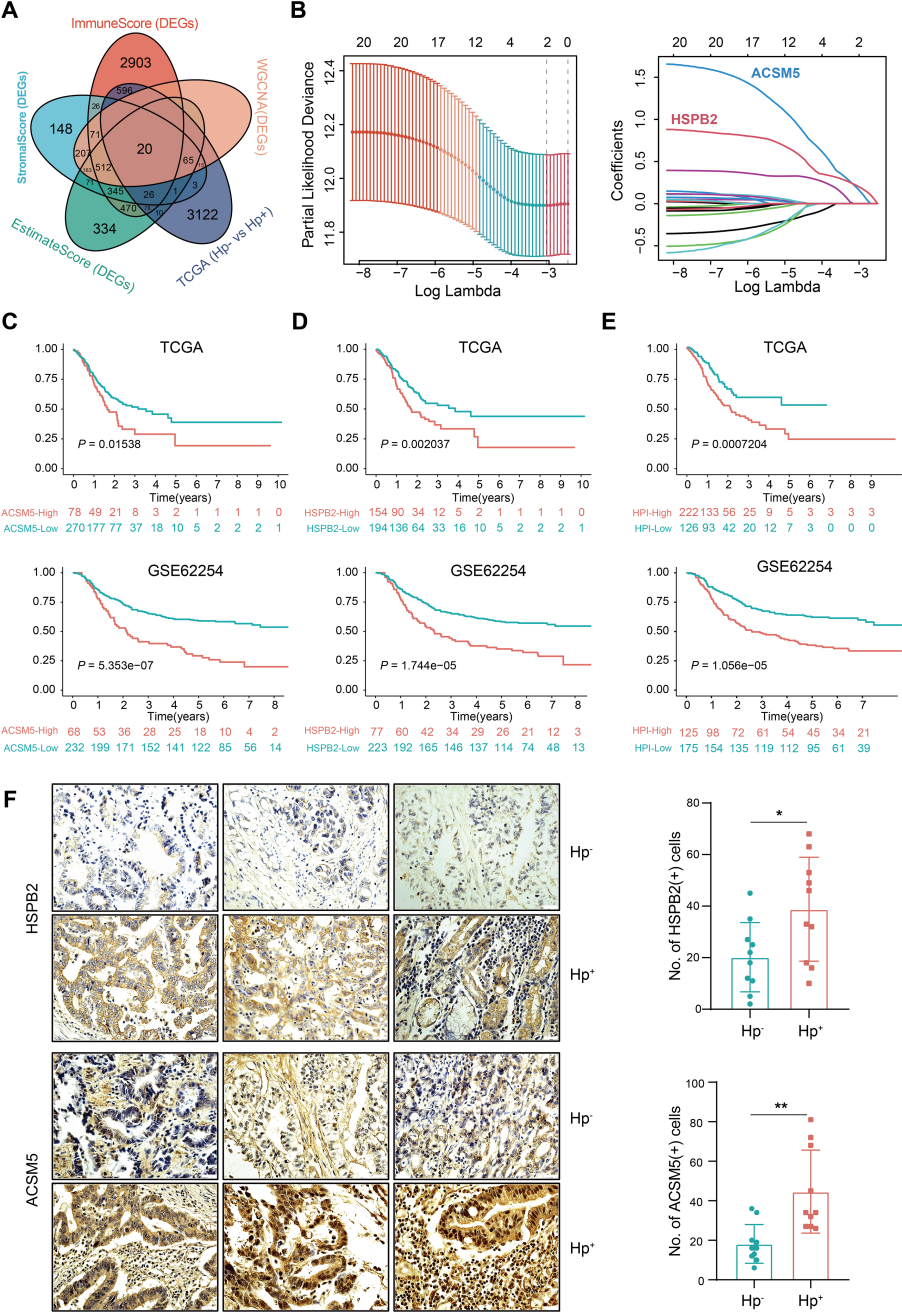
Development of the *H*. *pylori*-associated index and validation of its prognostic value. **(A)** Venn diagram shows the intersection of DEGs between the TME score, significant gene modules of WGCNA analysis and *H. pylori* infection groups in the TCGA. **(B)** LASSO analysis for identifying the most important genes. The minimal lambda value of HSPB2 is 0.1874 and ACSM5 is 0.2132. **(C)** Overall survival of ACSM5 expression groups in the TCGA and GSE62254 datasets. **(D)** Overall survival of the HSPB2 expression groups in the TCGA and GSE62254 datasets. **(E)** Overall survival of the low and high HPI groups in the TCGA and GSE62254 datasets. **(F)** Immunohistochemical staining of ACSM5 and HSPB2 on HP negative and positive patients. ns, not significant; *P < 0.05; **P < 0.01.

We further probed the TME score’s relationship with clinical characteristics, particularly *H. pylori* infection status, applying weighted gene co-expression network analysis (WGCNA). The optimal soft threshold of WGCNA was determined as 6 (**Supplementary Figure 2A**). We identified gene sets associated with pink, turquoise, yellow, and gray modules based on TME score correlations with *H. pylori* infection status (**Figure 1A**). Correlation analyses indicated these integrated modules had robust positive links with TME scores and *H. pylori* infection status (**Figure 1B**, **Supplementary Figure 2B**). In detail, the pink and turquoise modules were positively correlated, whereas yellow and gray modules showed negative associations with TME scores and *H. pylori* infection status (**Figure 1C**, **Supplementary Figure 2C**). When crossing DEGs from WGCNA, TCGA, and TME scores, we then singled out 20 genes indicative of their collective relevance to TME scores and *H. pylori* infection status (**Figure 2A**). Following LASSO Cox regression analysis, heat shock protein B2 (HSPB2) and acyl-CoA synthetase medium-chain family member 5 (ACSM5) were identified as the most significant genes associated with tumor microenvironment (TME) alterations and Helicobacter pylori infection. These genes were selected based on their coefficients corresponding to the minimal lambda value in the LASSO model, with HSPB2 and ACSM5 exhibiting values of 0.1874 and 0.2132, respectively (**Figure 2B**).

### Establishment and validation of a prognostic index for TME and *H. pylori* infection

To reveal the relationship of ACSM5 and HSPB2 with the prognosis of GC patients, a TME based prognostic index was developed with the following formula: HPI = 0.1874*ACSM5 + 0.2132*HSPB2. This index stratifies GC patients into high- and low-HPI groups based on an optimal cutoff value of 1.077131. We observed that *H. pylori*-infected patients, exhibiting higher ACSM5 and HSPB2 expression (**Figure 2F**, **Supplementary Figure 3**), faced inferior overall survival (OS) outcomes (**Figures 2C, D**). Moreover, patients in the high-HPI group presented with poorer OS (**Figure 2E**). Assessing the HPI’s relationship with clinical attributes, we revealed that patients afflicted with *H. pylori* infection, alongside higher T grades or stages, had a dismal prognosis (**Supplementary Figure 3**), reinforcing the HPI’s utility in outcome prediction. We then corroborated the efficacy and universality of the HPI through univariate and multivariate Cox regression analyses highlighting the HPI as a robust, independent prognostic factor in forecasting OS (**Supplementary Figure 4**). These results solidified both individual genes and the composite HPI as accurate prognosticators of GC patient outcomes.

### The *H. pylori*-associated prognostic index predicts therapeutic effects

**Figure 3:**
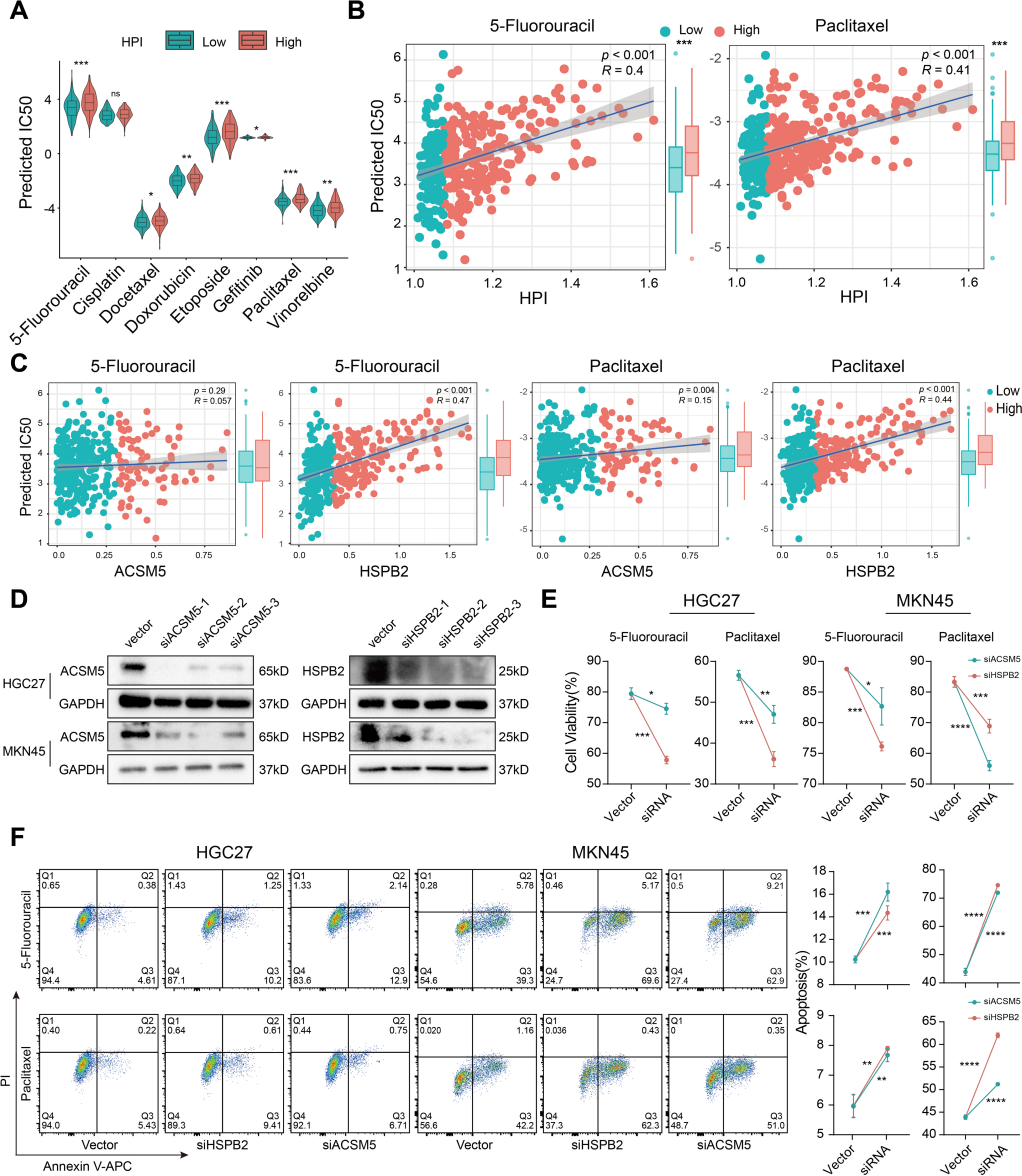
*H*. *pylori* infection induces drug resistance based on the HPI. **(A)** Comparison of the distribution of IC50 values for common chemotherapy drugs between the low- and high-HPI groups. **(B)** Association between the HPI and predicted IC50 values of 5-fluorouracil and paclitaxel. **(C)** Relationship of the expression of ACSM5 and HSPB2 with the predicted IC50 values of 5-fluorouracil and paclitaxel. **(D)** Western blotting was used to verify the gene knockout efficiency of ACSM5 and HSPB2 siRNA in HGC-27 and MKN45 gastric cancer cell lines. **(E)** Cell viability assessment after siACSM5 and HSPB2 in HGC-27 and MKN45 gastric cancer cell lines treated with 5-fluorouracil and paclitaxel. **(F)** Apoptosis rate detection after siACSM5 and HSPB2 in HGC-27 and MKN45 gastric cancer cell lines treated with 5-fluorouracil and paclitaxel. The Wilcoxon test was used to calculate the significant difference between two groups. ns, not significant; *P < 0.05; **P < 0.01; ***P < 0.001, ****P < 0.0001.

Chemotherapy remains a crucial component in extending the survival of gastric cancer patients. Whether *H. pylori* infection affects the chemotherapy response of patients has yet to be fully understood. Here, we computed IC50 values for several commonly anticancer drugs based on the HPI (**Figure 3A**). We revealed that patients with lower HPIs were more responsive to numerous anticancer agents, including 5-fluorouracil, docetaxel, doxorubicin, etoposide, gefitinib, paclitaxel and vinorelbine, implying that patients with low HPIs may achieve better chemotherapy outcomes than those with high HPIs. The high-HPI group showed less efficacy to 5-fluorouracil and paclitaxel, the commonly used drugs for gastric cancer treatment (**Figure 3B**).

Given that the HPI is contingent upon ACSM5 and HSPB2 expression, and we previously found drug resistance in the high-HPI group, we also noticed that a positive association between these genes’ expression and resistance to key therapies like 5-fluorouracil and paclitaxel (**Figure 3C**). We subsequently verified the efficacy of siRNA with western blotting experiments and selected the best siRNA for subsequent drug sensitivity experiments in HGC-27 and MKN45 cells (**Figure 3D**). Compared with controls, cells transfected with siRNA demonstrated heightened sensitivity to therapeutic agents and increased rates of apoptosis (**Figures 3E, F**), signifying that silencing HSPB2 and ACSM5 augmented drug susceptibility. The results implied that *H. pylori* infection-associated genes not only affect the efficacy of conventional chemotherapy agents but also contribute to suboptimal patient outcomes.

### The *H. pylori*-associated prognostic index was related to immune cell infiltration

**Figure 4:**
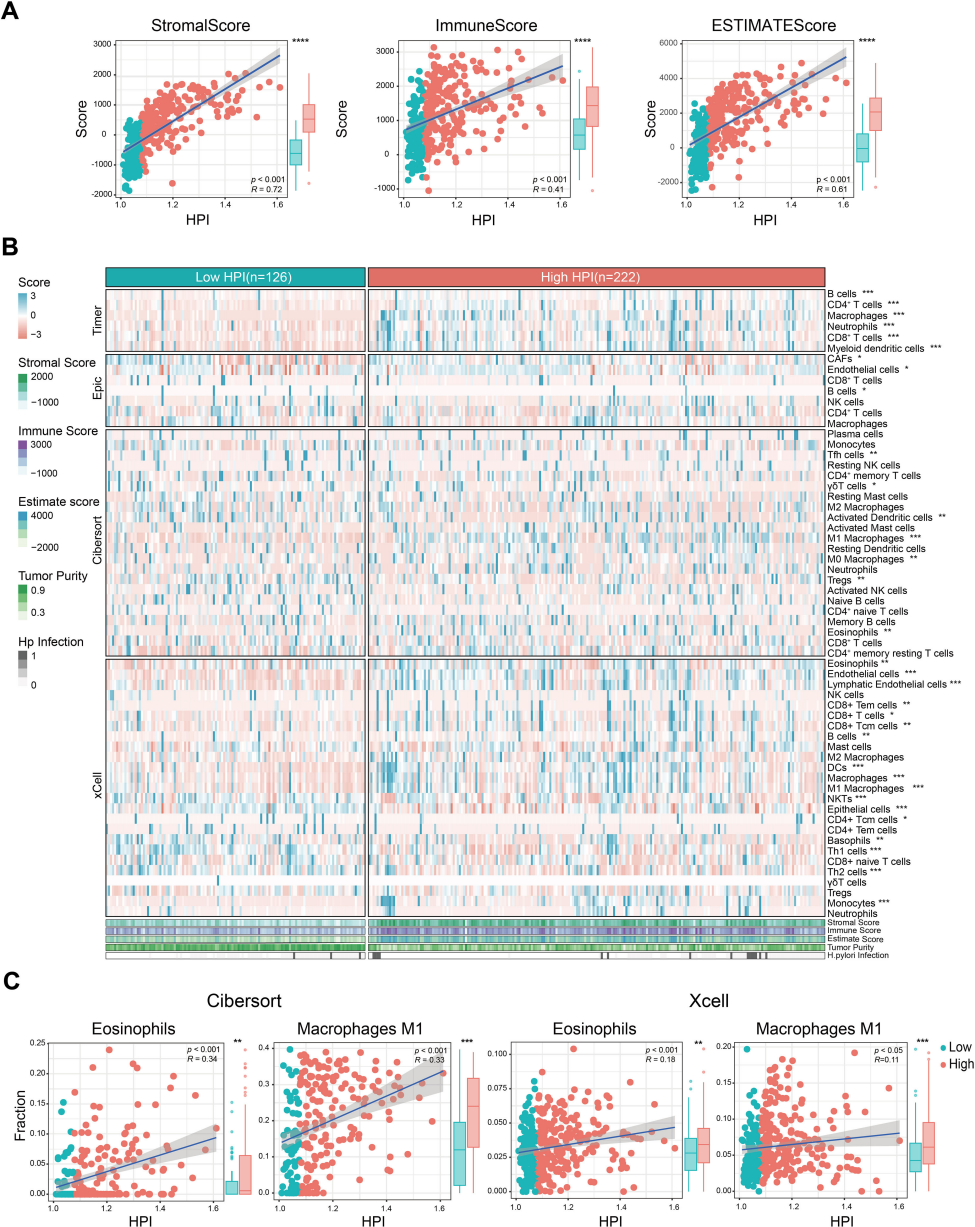
Correlation between TME features and *H*. *pylori* infection. **(A)** Relationship of TME scores and tumor purity with HPI. **(B)** Heatmap of marker expression for tumor microenvironment-associated cells in the different HPI groups. The TME scores, tumor purity and *H*. *pylori* infection status are also illustrated under the heatmap. **(C)** Association between the HPI and the infiltration of macrophages/eosinophils predicted by CIBERSORT and the xCell algorithm. The Wilcoxon test was used to assess the significance of differences between two groups. *P < 0.05; **P < 0.01; ***P < 0.001, ****P < 0.0001.

Tumor microenvironment remodeling can induce chemotherapy response and confer drug resistance in gastric cancer patients (25). Confirming this, we found the HPI satisfyingly correlated with higher TME scores (**Figure 4A**). Human leukocyte antigens (HLAs), an independent factor for tumor-associated antigen presentation, play a critical role in the antitumor immune response and neoplastic tumor progression (26). Immune checkpoints, as regulators of T cells, can reflect T cell exhaustion in the TME (27). We assessed the expression of 24 HLA family genes and 48 immune checkpoint genes across patient specimens, revealing that 18 HLA family genes and 37 immune checkpoint genes were upregulated in the high-HPI subgroup (**Supplementary Figure 5**). Classic T cell exhaustion markers, including PD-1, CTLA-4, TIM-3, TIGHT, BTLA, and LAG3, were found at elevated levels in the high-HPI group, implicating their role in the exacerbated clinical outcomes associated with *H. pylori* infection.

To validate the main cell infiltration involved, we estimated the infiltration levels using various algorithms (TIMER, EPIC, CIBERSORT and xCell). There was a clear differential expression among stromal cells, notably cancer associated fibroblast (CAFs), endothelial cells, and epithelial components (**Figure 4B**). The high-HPI group exhibited increased infiltration of CAFs and endothelial cells in comparison to epithelial cells. Furthermore, adaptive immune T cell subsets, B cell and innate immune cells like M0 and M1 macrophages, alongside eosinophils, were enriched in the high-HPI cohort (**Figure 4C**).

### Myeloid immune cell infiltration in *H. pylori* infected gastric cancer

**Figure 5:**
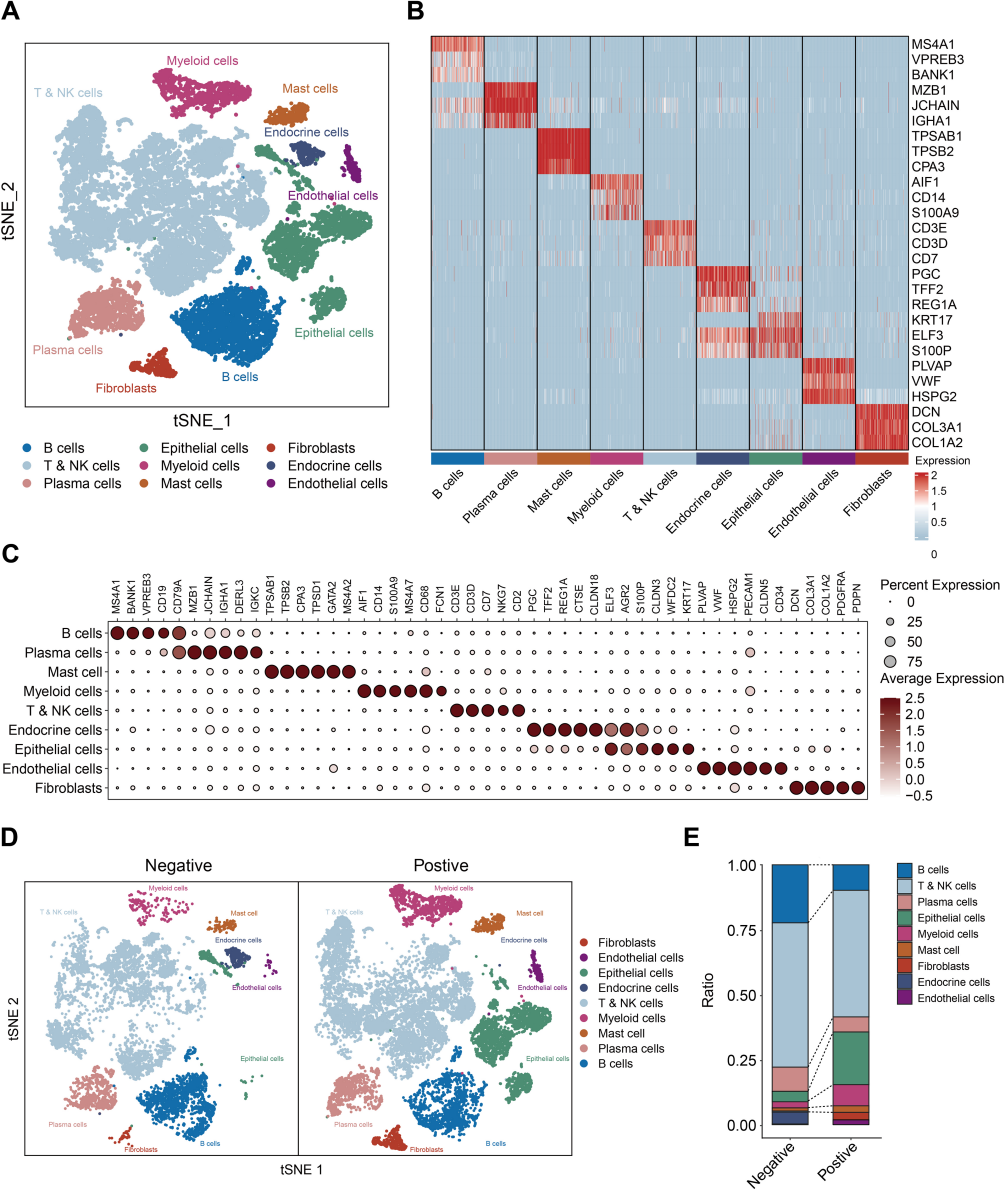
Profiling the the gastric cancer tumor microenvironment at single-cell level. **(A)** t-Stochastic Neighbor Embedding (tSNE) of 16653 single cells from 6 patients, allowing the visualization of 9 clusters. **(B)** Heatmap showing the differentially expressed genes for each cluster. **(C)** Dot plots showing marker genes. **(D)** t-Stochastic Neighbor Embedding (tSNE) of the **(H)** pylori negative and positive samples. **(E)** the cell ratio of *H. pylori* negative and positive samples.

Reaffirming previous findings, we reanalyzed single-cell profiles from selected HP-negative and positive samples. t-Stochastic Neighbor Embedding (tSNE) discerned nine cellular clusters, including T and NK cells, B cells, plasma cells, myeloid cells, mast cells, endothelial cells, fibroblast cells, epithelial cells, and endocrine cells (**Figures 5A–C**). In addition, we compared the cell distribution based on the *H. pylori* infected status. The distribution of myeloid and mast cells markedly varied based on *H. pylori* status (**Figures 5D, E**), profound heterogeneity in *H. pylori*-infected gastric cancer.

**Figure 6:**
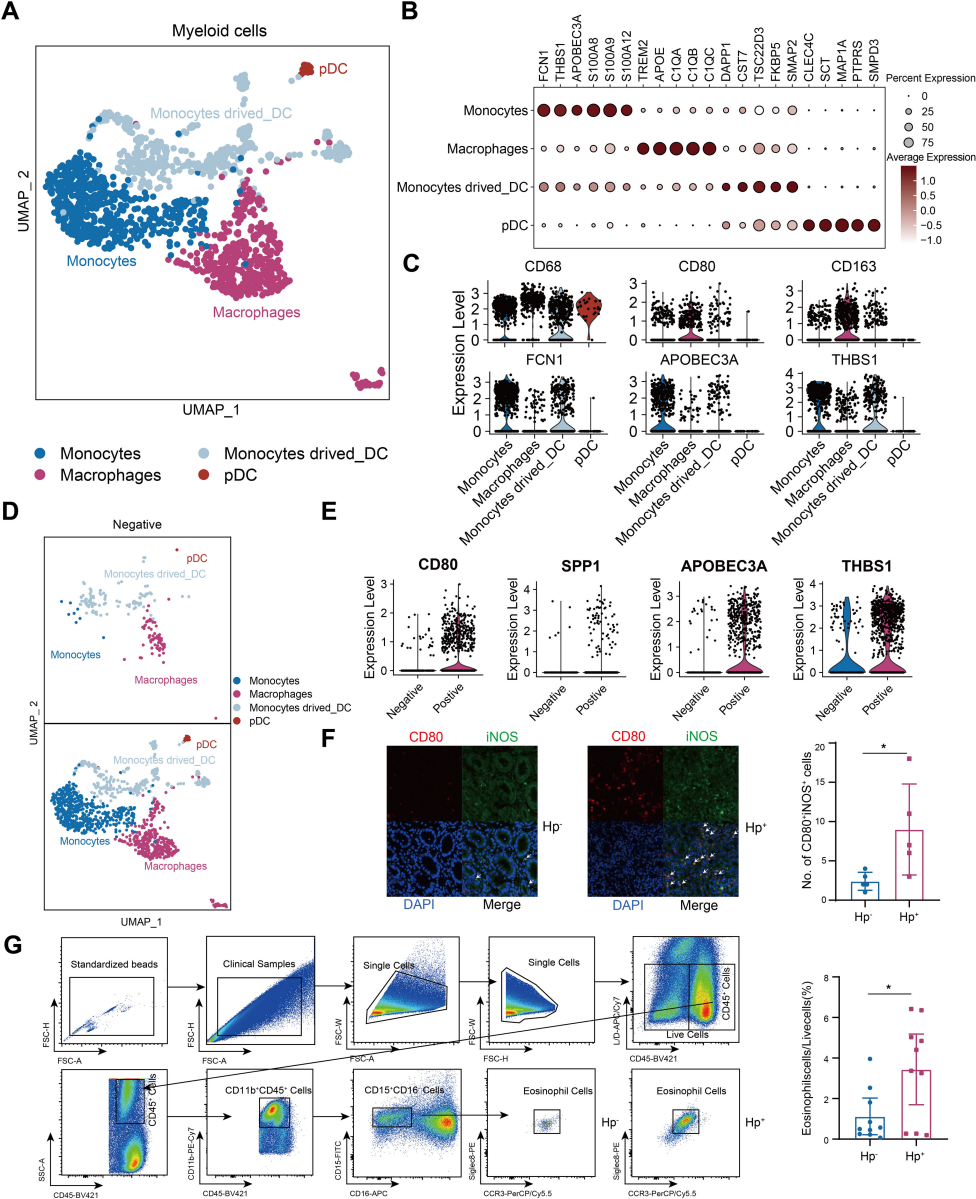
*H*. *pylori^+^* samples withe more myeloid immune cell infiltration. **(A)**t-Stochastic Neighbor Embedding (tSNE) of the reclustered myeloid cells. **(B)** Dot plots showing marker genes. **(C)** Violin plots of each cluster. **(D)** t-Stochastic Neighbor Embedding (tSNE) of the *H*. pylori negative and positive samples in myeloid cells. **(E)** Violin plots of *H*. pylori negative and positive samples in myeloid cells. **(F)** The distribution of macrophages was evaluated in *H*. *pylori^-^* and *H*. *pylori^+^* samples by immunofluorescence (n=5, respectively). **(G)** The gating strategy and the number of eosinophils in *H*. *pylori^-^* and *H*. *pylori^+^* samples as determined by flow cytometry (n=10, respectively). The T-test was used to assess the significance of differences between two groups. *P < 0.05.

Given that the myeloid composition was profoundly altered in HP infected tumors, we identified 4 myeloid subpopulations: monocytes, macrophages, monocytes derived dendritic cells and plasmacytoid dendritic cells (pDC) (**Figures 6A–C**). Monocytes and macrophages dominated in *H. pylori*-infected samples (**Figure 6D**), with monocytes predominantly expressing APOBEC3A and THBS1, while macrophages were characterized by CD80 and SPP1 (**Figure 6E**). Consistent with studies linking alterations in B-cell, LAG3-expressing T-cell, dendritic cell and macrophage (TAM) infiltration with chemotherapy insensitivity (21, 26, 28), we found increased macrophage infiltration in *H. pylori^+^* GC specimens (**Figure 6F**). *H. pylori* infection been documented to induce eosinophil accumulation (29), implicated in gastritis in murine models (30). Despite the focus on eosinophils in allergic conditions, their role in cancer remains underexplored. We verified their heightened presence in *H. pylori*-infected GC specimens (**Figure 6G**). These findings underscored the variance in TME components and their contribution to the more severe pathology in *H. pylori*-infected GC patients.

## Discussion

*H. pylori* infection is recognized as the primary risk factor implicated in the etiology of gastric cancer, where it disrupts cellular signaling and engenders chronic inflammation of the gastric mucosa, thereby remodeling the local microenvironment (2). In this study, we first reported that TME scores are elevated in *H. pylori^+^* GC patients, correlating with poorer clinical outcomes. We developed the *Helicobacter pylori* Prognostic Index (HPI) to evaluate its influence on TME complexity in GC and demonstrated its prognostic significance. Unlike previous models, our index uniquely integrates *H. pylori*-associated gene expression with TME-related features, thereby combining microbial infection status with immune infiltration signatures. This dual-layered approach offers a novel prognostic tool that simultaneously captures microbial and immunological dynamics in GC. Furthermore, our findings indicate that *H. pylori* infection alters the composition of immune and stromal cell populations within the TME, which may contribute to the development of chemoresistance. Drug therapy remains a cornerstone in the treatment of GC. In this study, we first observed elevated expression levels of ACSM5 and HSPB2 in Helicobacter pylori-positive GC patients compared to those without the infection. The expression of ACSM5 has been previously linked to tumor aggressiveness and poor prognosis (31), and its role in fatty acid metabolism suggests it may contribute to tumor energy homeostasis and survival (32). In parallel, HSPB2 overexpression has been implicated in the inhibition of apoptosis by suppressing the extrinsic apoptotic pathway-specifically through inhibition of apical caspase-8 and -10 activation-thus preventing Bid cleavage and subsequent caspase-3 activation, conferring resistance to TRAIL- and TNF-α-induced apoptosis (33). Together, these findings suggest that ACSM5 and HSPB2 may serve as prognostic biomarkers and potential therapeutic targets in GC. Consistently, our functional experiments demonstrated that silencing ACSM5 and HSPB2 increased the sensitivity of GC cells to standard chemotherapeutic agents, further supporting their involvement in drug resistance mechanisms. Moreover, we detected the upregulation of ACSM5 and HSPB2 in *H. pylori^+^* GC patients, underscoring the need for tailored therapeutic strategies for *H. pylori*-infected GC patients based on ACSM5 and HSPB2 expression levels. In addition, the prognosis and immunotherapy outcome of patients with *H. pylori* infection is controversial (34–37), implying an intricate microenvironmental components warrants further investigation.

**Figure 7:**
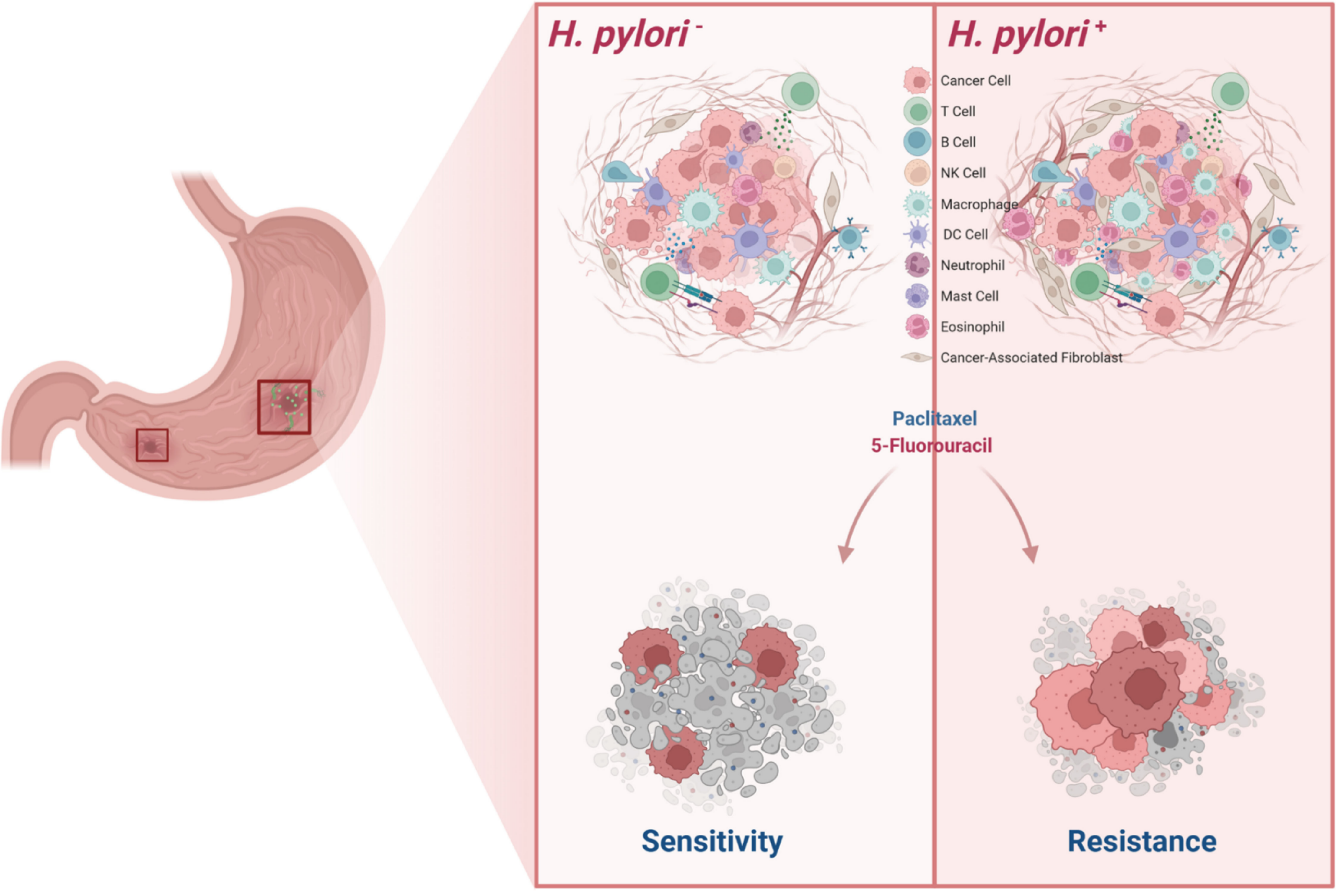
Schematic diagram of *H. pylori* induces TME remodeling and chemoresistance in gastric cancer.

*H. pylori* infection plays a pivotal role in reshaping the TME of GC, fostering dynamic interactions among diverse stromal and immune cell populations. Notably, patients with a high HPI exhibited marked infiltration of CAFs, endothelial cells, and both innate and adaptive immune cell subsets. The *H. pylori*-driven transition of fibroblasts into CAFs is not only a key event in promoting tumor invasion and chemoresistance (38–40), but also contributes significantly to the orchestration of angiogenesis and the recruitment of immune cells, thereby sustaining chronic inflammation and tumor progression (41–47). Among the recruited immune populations, tumor-associated macrophages (TAMs) emerge as central mediators of immunosuppression within the gastric TME, particularly in the context of chronic *H. pylori*-induced gastritis (21). TAMs secrete a repertoire of cytokines—including TNF-α, IL-1β, IL-4, IL-10, and IL-13-that collectively promote tumorigenesis (42) and induce T-cell dysfunction, in part through the upregulation of immune checkpoint molecules such as PD-L1 (28, 48). In addition, TAMs facilitate endothelial cell activation and survival by releasing pro-inflammatory and pro-angiogenic factors, further supporting neovascularization and tumor progression (49). Notably, previous studies have demonstrated that *H. pylori* CagA-positive strains can activate the NF-κB signaling pathway and induce the release of pro-inflammatory cytokines, which may contribute to the polarization of macrophages toward an M1-like phenotype (46). Eosinophils have also been reported to increase in *H. pylori*-infected gastric mucosa (29), and evidence from murine tumor models suggests that eosinophils preferentially infiltrate hypoxic regions of tumors (50, 51). Activated eosinophils are capable of secreting proangiogenic factors *in vitro (* 52), although their angiogenic functions *in vivo* remain less well defined (49). Additionally, eosinophils have been shown to promote tumor cell migration and bone metastasis via the CCL6–CCR1 signaling axis, and inhibition of this pathway significantly reduces eosinophil-mediated tumor dissemination (53). Moreover, eosinophil-derived Charcot-Leyden crystal protein/galectin-10 (CLC-P/Gal10) has been implicated in chemoresistance in mesothelioma, where elevated expression correlates with poor prognosis; importantly, anti-eosinophil therapies have been shown to restore chemosensitivity in preclinical models (54). Collectively, these findings highlight that tumor-infiltrating myeloid cells-including macrophages and eosinophils-can secrete CC-chemokine ligands, receptors, and various cytokines to regulate the tumor microenvironment and promote angiogenesis (49). In our study, single-cell RNA sequencing revealed increased infiltration of both macrophages and eosinophils in patients with high HPI scores, a trend that was especially prominent in *H. pylori*-positive gastric cancer cases. These results suggest that remodeling of the tumor microenvironment in *H. pylori*-infected GC contributes to increased immune cell complexity and may ultimately lead to enhanced chemoresistance (**Figure 7**).

This study establishes the HPI as a substantive tool for assessing the impact of *H. pylori* infection on the TME in GC and highlights the potential therapeutic relevance of targeting macrophages and eosinophils in *H. pylori*-positive GC. While our findings offer valuable insights, we acknowledge several limitations. First, the availability of patient data specifying *H. pylori* infection status was limited, which may constrain the generalizability of our conclusions. Second, the large number of DEGs identified may have inadvertently excluded other relevant candidates—such as TP53, a well-established regulator of the TME (10, 55, 56). We also acknowledge that the relatively small cohort used for single-cell RNA sequencing may limit the generalizability of the observed immune cell distribution patterns. Although our analysis provides preliminary insight into *H. pylori*-associated myeloid remodeling at single-cell resolution, validation in larger, independent patient cohorts is necessary to substantiate and extend these findings. Moreover, while we observed a strong association between high HPI scores and increased infiltration of macrophages and eosinophils, we did not independently assess the correlation between individual gene expression levels of HSPB2 or ACSM5 and specific immune cell populations. Future studies will aim to elucidate the distinct contributions of these genes to immune remodeling in *H. pylori*-infected GC. Therefore, further investigation is warranted to validate the prognostic accuracy and clinical utility of the HPI in comparison with established biomarkers, particularly in the context of predicting immunotherapy response in GC. Our findings underscore the translational potential of the HPI in refining prognostic stratification and informing individualized therapeutic strategies for *H. pylori*-positive GC patients. These results also reinforce the broader clinical imperative of *H. pylori* eradication as part of comprehensive GC management.

In summary, the HPI, constructed from newly identified and complementary biomarkers, demonstrates a strong association with the tumor microenvironment and serves as a robust predictor of prognosis in gastric cancer patients. Comprehensive analyses of the immune microenvironment, along with drug resistance profiling and validation in both clinical samples and cell line models, underscore the clinical utility of integrating biomarker and immune cell assessment. These findings suggest that HPI-guided stratification may enhance the precision of therapeutic strategies and ultimately improve clinical outcomes in gastric cancer management.

## Data Availability

The original contributions presented in the study are included in the article/**Supplementary Material**. Further inquiries can be directed to the corresponding authors.

## Supplementary material

Supplementary Figure 1 TME scores are associated with *H. pylori* infection status and the outcome of GC patients. **(A)** Difference analysis of the distribution of TME scores and tumor purity in *H. pylori^-^* and *H. pylori^+^* CG patients. **(B)** Survival analysis based on the best cutoff for stromal score. **(C)** Survival analysis based on the best cutoff for immune score. **(D)** Survival analysis based on the best cutoff for estimate score. **(E)** Correlation between stromal score and tumor purity according to “estimate” algorithms. The Wilcoxon test was used to assess the significance of differences between two groups. ns not significant; *P < 0.05; **P < 0.01; ***P < 0.001.

Supplementary Figure 2 Determination of the soft threshold power and the correlations among functional modules **(A)** Function distribution of the scale free fit index (y-axis, left) and mean connectivity (y-axis, right) with the soft threshold power (x-axis). The cutoff value of the scale-free fit index is 0.9 (red line). **(B)** Correlation between significant module membership and immune/estimate score. **(C)** Correlation between membership in a single significant module (gray, pink, turquoise and yellow modules) and immune/estimate scores.

Supplementary Figure 3 Comparison of clinical feature distribution in different HPI groups. The Wilcoxon test was used to assess the significance of differences between two groups. ns not significant; *P < 0.05; **P < 0.01; ***P < 0.001.

Supplementary Figure 4 Assessment of the *H. pylori*-associated prognosis index. **(A, B)** Forest plot of the univariate and multivariate analyses of clinical features and HPI in the TCGA and GSE62254 datasets. **(C, D)** Time-dependent receiver operator characteristic (ROC) value in the TCGA and GSE62254 datasets.

Supplementary Figure 5 Comparison of the distribution of HLA family- and immune checkpoint-associated genes in the low- and high-HPI groups. The Wilcoxon test was used to assess the significance of differences between two groups. ns not significant; *P < 0.05; **P < 0.01; ***P < 0.001.
