# Supplementary figures and images for: Transcriptional profiling reveals H.pylori-associated genes induced inflammatory cell infiltration and chemoresistance in gastric cancer
Source: Front Immunol. 2025 May 30;16:1592558. doi: 10.3389/fimmu.2025.1592558 (PMC12162319; doi:10.3389/fimmu.2025.1592558)

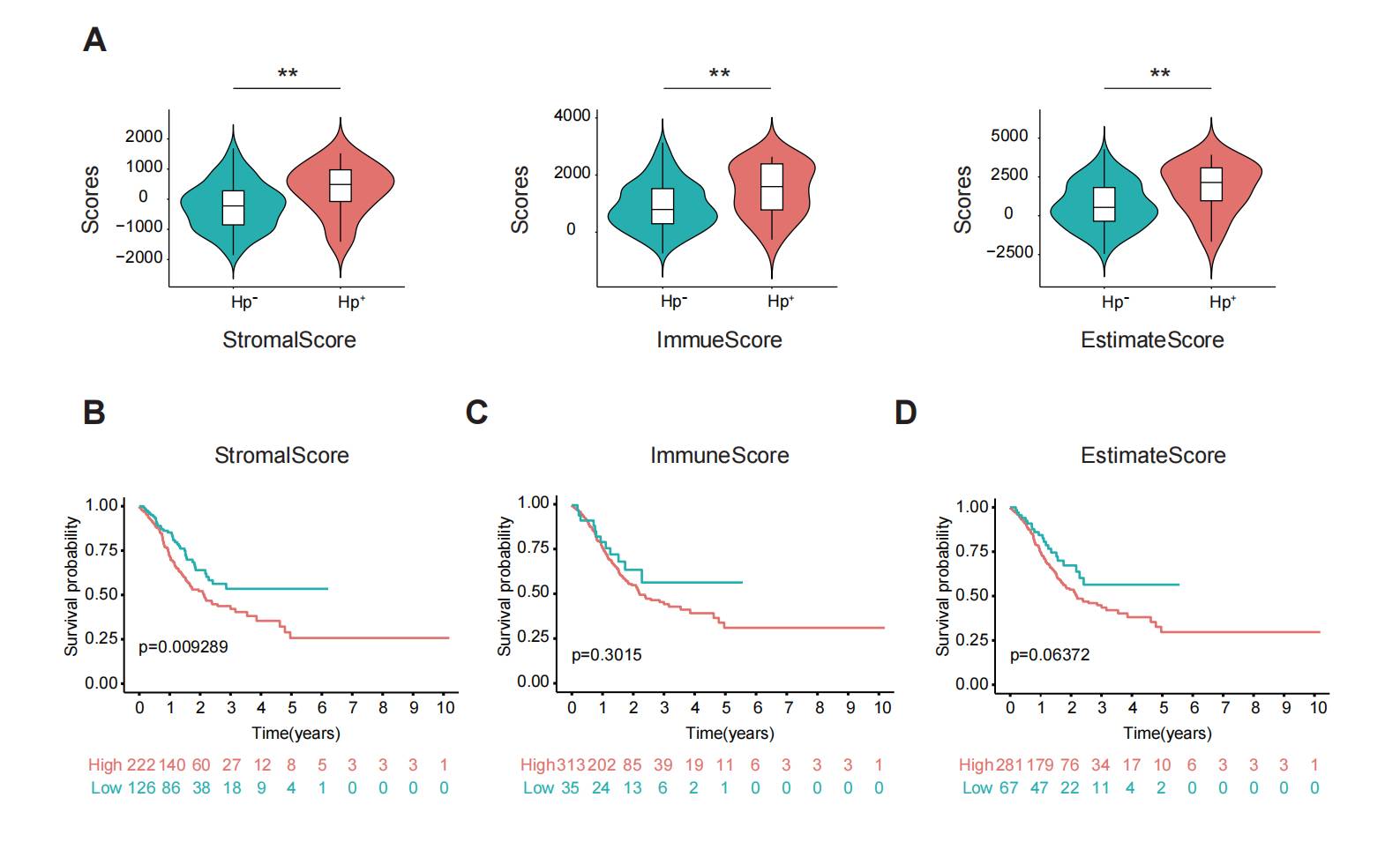

Supplement: Supplementary Figure 1 — TME scores are associated with H. pylori infection status and the outcome of GC patients. (A) Difference analysis of the distribution of TME scores and tumor purity in H. pylori- and H. pylori+ CG patients. (B) Survival analysis based on the best cutoff for stromal score. (C) Survival analysis based on the best cutoff for immune score. (D) Survival analysis based on the best cutoff for estimate score. (E) Correlation between stromal score and tumor purity according to “estimate” algorithms. The Wilcoxon test was used to assess the significance of differences between two groups. ns not significant; *P < 0.05; **P < 0.01; ***P < 0.001. [file Image1.jpeg]

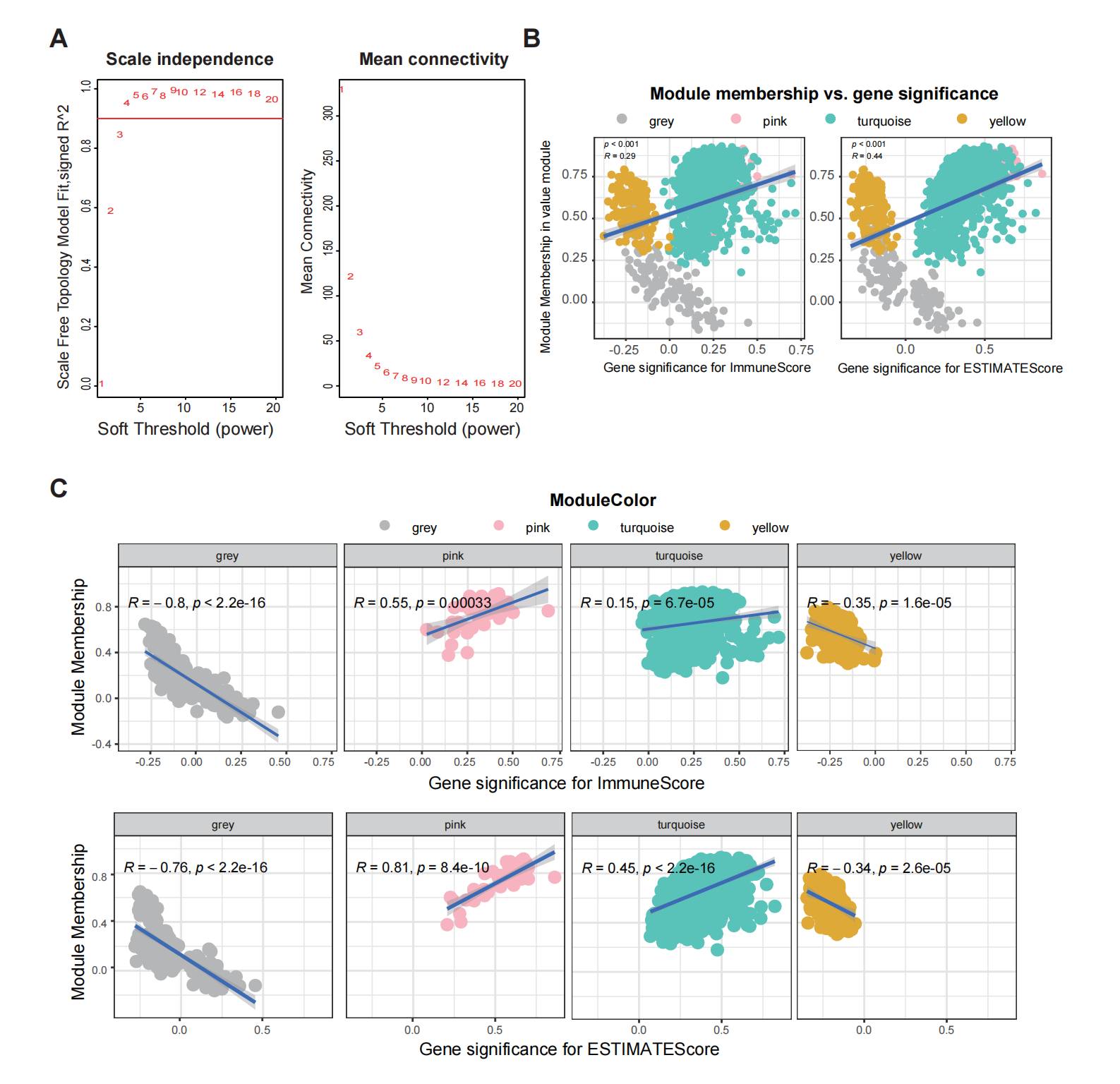

Supplement: Supplementary Figure 2 — Determination of the soft threshold power and the correlations among functional modules (A) Function distribution of the scale free fit index (y-axis, left) and mean connectivity (y-axis, right) with the soft threshold power (x-axis). The cutoff value of the scale-free fit index is 0.9 (red line). (B) Correlation between significant module membership and immune/estimate score. (C) Correlation between membership in a single significant module (gray, pink, turquoise and yellow modules) and immune/estimate scores. [file Image2.jpeg]

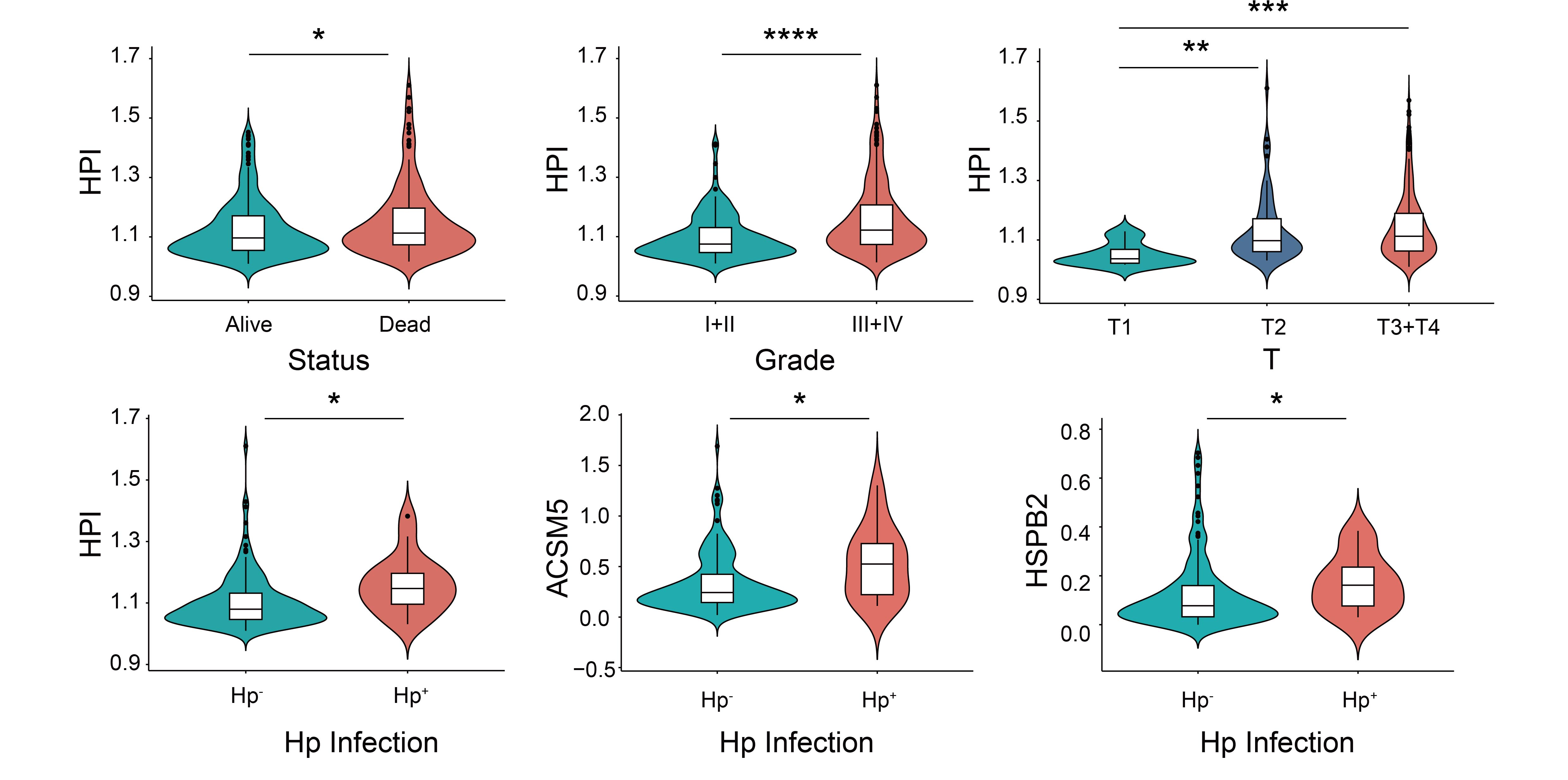

Supplement: Supplementary Figure 3 — Comparison of clinical feature distribution in different HPI groups. The Wilcoxon test was used to assess the significance of differences between two groups. ns not significant; *P < 0.05; **P < 0.01; ***P < 0.001. [file Image3.jpeg]

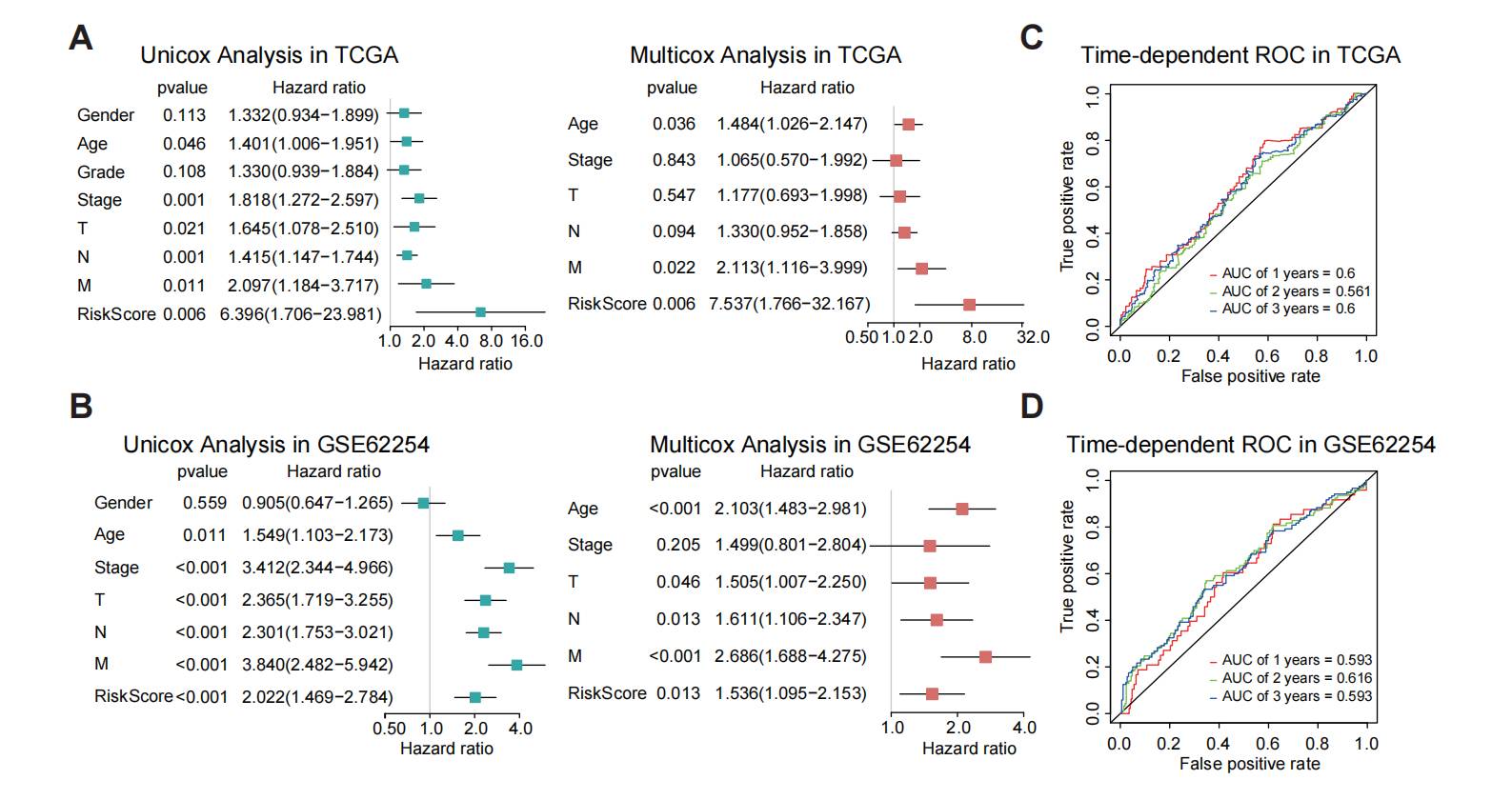

Supplement: Supplementary Figure 4 — Assessment of the H. pylori-associated prognosis index. (A, B) Forest plot of the univariate and multivariate analyses of clinical features and HPI in the TCGA and GSE62254 datasets. (C, D) Time-dependent receiver operator characteristic (ROC) value in the TCGA and GSE62254 datasets. [file Image4.jpeg]

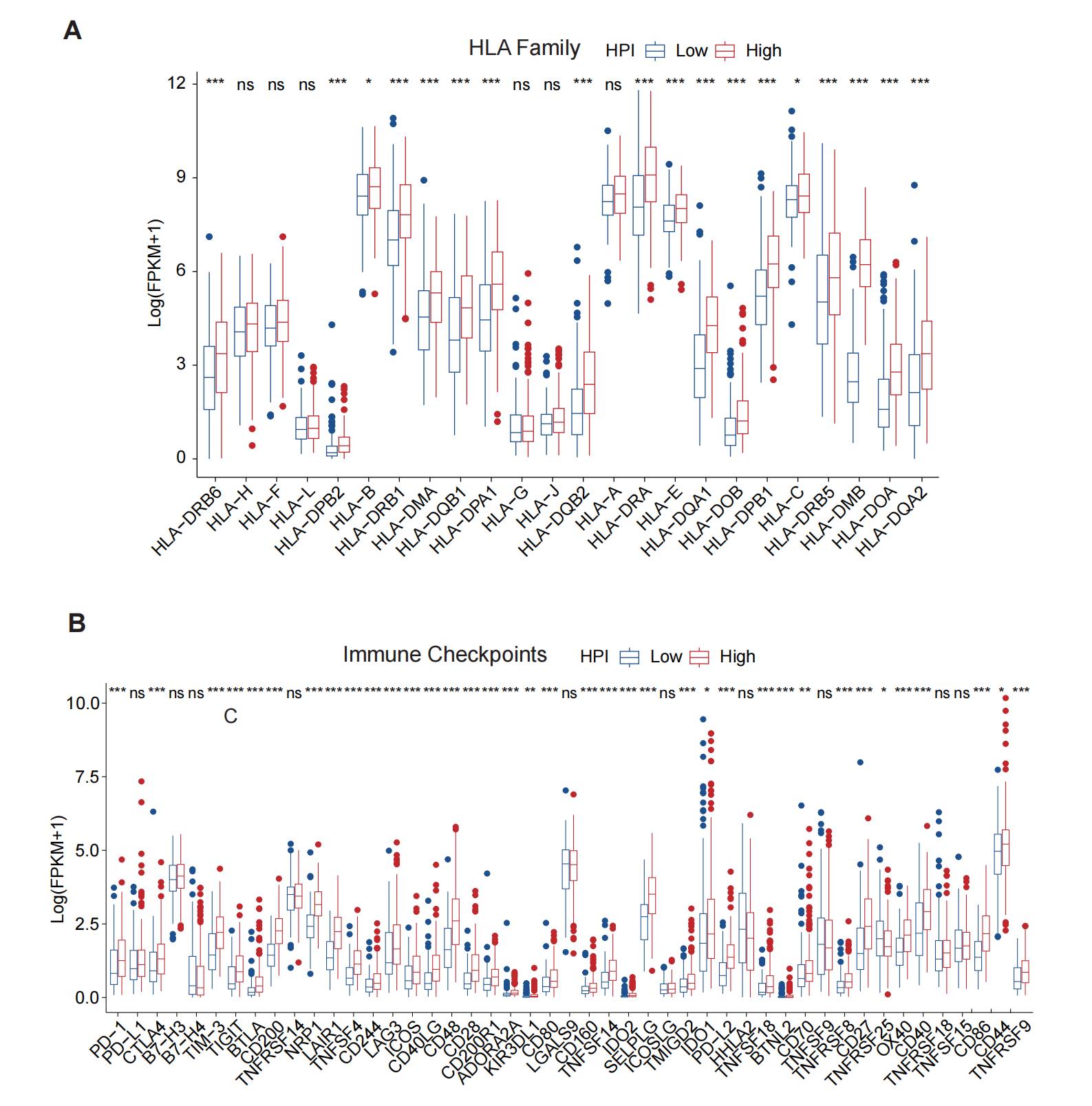

Supplement: Supplementary Figure 5 — Comparison of the distribution of HLA family- and immune checkpoint-associated genes in the low- and high-HPI groups. The Wilcoxon test was used to assess the significance of differences between two groups. ns not significant; *P < 0.05; **P < 0.01; ***P < 0.001. [file Image5.jpeg]

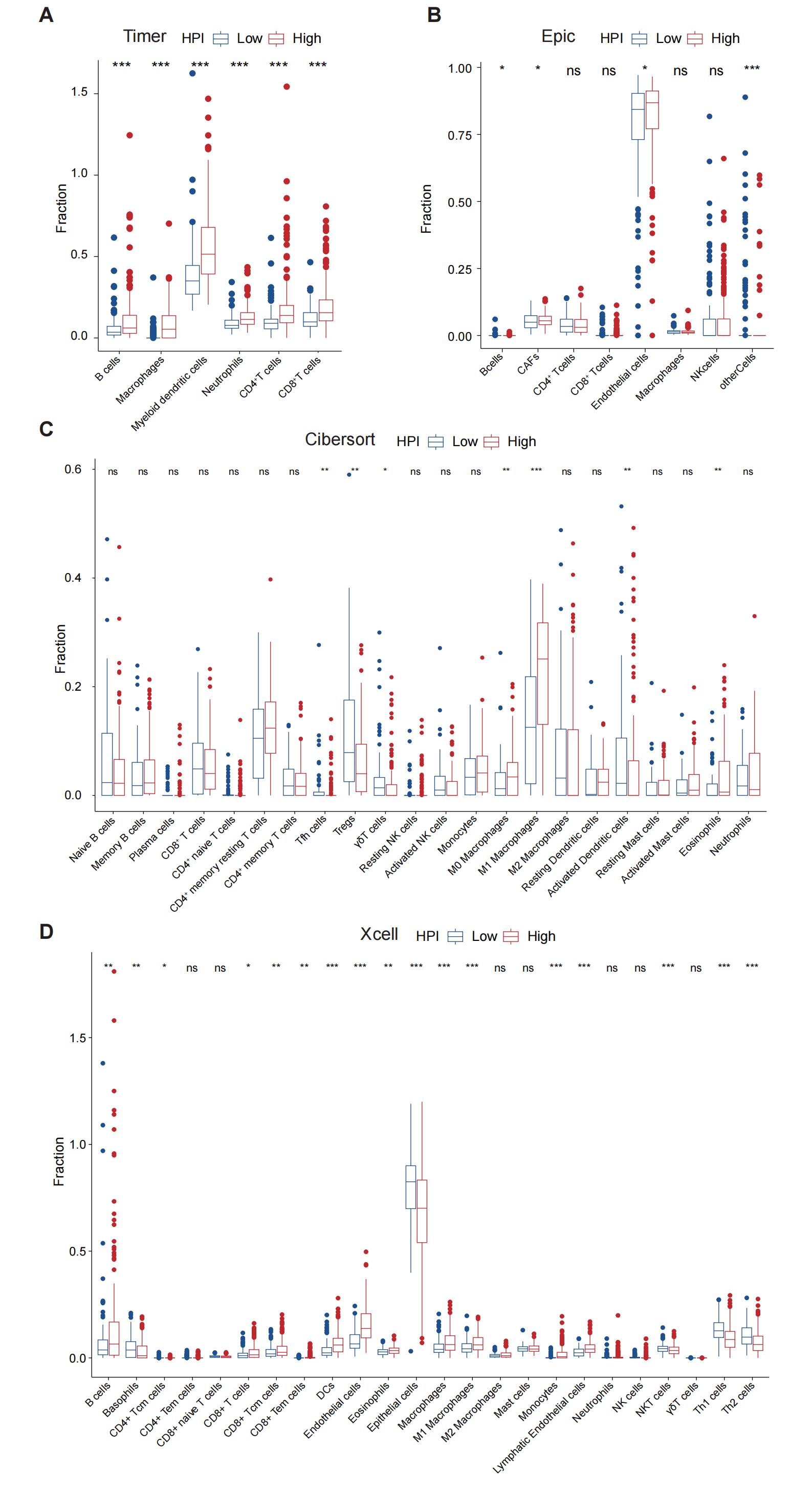

Supplement: Supplementary file 6 [file Image6.jpeg]
